# Comparative performance of traditional and novel adiposity indices for predicting insulin resistance and metabolic syndrome in Chinese women with polycystic ovary syndrome

**DOI:** 10.3389/fnut.2026.1835729

**Published:** 2026-06-15

**Authors:** Huichao Qin, Baichao Shi, Yabing Li, Fengjuan Lu, Muxin Guan, Jiannan Yu, Zhuwei Gao, Mengyi Zhu, Yang Liu, Hang Ge, Xiaoke Wu, Yuehui Zhang

**Affiliations:** 1 Heilongjiang University of Chinese Medicine, Harbin, China; 2 Heilongjiang Provincial Hospital, Harbin, China; 3 Zhejiang Provincial Hospital of Chinese Medicine and Zhejiang Chinese Medical University, Hangzhou, China; 4 The First Affiliated Hospital, Heilongjiang University of Chinese Medicine, Harbin, China

**Keywords:** adiposity index, insulin resistance, metabolic syndrome, polycystic ovary syndrome, predictive performance

## Abstract

**Objective:**

This study aimed to comprehensively evaluate and compare the predictive performance of seven adiposity indices—both traditional (waist-to-hip ratio, WHR; waist-to-height ratio, WHtR) and novel (visceral adiposity index, VAI; Chinese visceral adiposity index, CVAI; lipid accumulation product, LAP; triglyceride-glucose index, TyG; cardiometabolic index, CMI)—for identifying insulin resistance (IR) and metabolic syndrome (MetS) in a large cohort of Chinese women with polycystic ovary syndrome (PCOS).

**Methods:**

This secondary analysis enrolled 944 Chinese women with PCOS from the PCOSAct trial. IR was defined as homeostasis model assessment of insulin resistance (HOMA-IR) ≥ 2.69, and MetS was diagnosed in accordance with Chinese female-specific criteria. Receiver operating characteristic (ROC) curve analysis was applied to assess the discriminatory ability of each adiposity index, and correlations between these indices and metabolic, reproductive, hepatic as well as renal parameters were analyzed.

**Results:**

The prevalence of IR and MetS in this cohort was 50 and 15.68%, respectively. All adiposity indices were significantly correlated with key metabolic parameters (all *p* < 0.001). For predicting IR, LAP (AUC = 0.815) and CVAI (AUC = 0.813) showed the highest diagnostic accuracy. For predicting MetS, CMI (AUC = 0.905), LAP (AUC = 0.898) and VAI (AUC = 0.893) presented the optimal predictive efficacy. Additionally, these adiposity indices were significantly correlated with sex hormone-binding globulin, free androgen index, liver enzymes and bilirubin levels.

**Conclusion:**

Novel adiposity indices, especially CVAI, LAP and CMI, are effective and non-invasive tools for the early identification of IR and MetS in Chinese women with PCOS. The integration of these indices into clinical practice can improve risk stratification and guide timely interventions for metabolic comorbidities in this high-risk population.

**Trial registration:**

ClinicalTrials.gov, identifier: NCT01573858; Chictr.org.cn, identifier: ChiCTR-TRC-12002081.

## 1 Introduction

Polycystic ovary syndrome (PCOS) is the most common reproductive, endocrine, and metabolic disorder in women of reproductive age, with a global prevalence of 9.2% (1). Beyond its core features of oligoovulation, hyperandrogenism, and polycystic ovarian morphology, PCOS is frequently accompanied by metabolic abnormalities: approximately 50% of PCOS patients are complicated by insulin resistance (IR), 20–40% by metabolic syndrome (MetS) (2). These metabolic abnormalities not only significantly increase the risk of type 2 diabetes mellitus and cardiovascular diseases but also exacerbate ovarian dysfunction and impair reproductive outcomes, thus becoming a major threat to the long-term health of PCOS patients.

Obesity, particularly abdominal adiposity, acts as a key driver of metabolic abnormalities in PCOS. By dysregulating proinflammatory cytokines (tumor necrosis factor-*α*, interleukin-6) and adipokines (leptin, adiponectin) secreted by adipose tissue, abdominal adiposity further impairs insulin signaling pathways, thereby inducing IR and triggering subsequent metabolic sequelae (3, 4). Body mass index (BMI) is a conventional anthropometric indicator that is easy to apply in clinical practice; however, it only reflects overall body mass and cannot distinguish fat distribution or visceral adiposity (5), limiting its value for predicting metabolic risk in PCOS populations. While waist circumference (WC) can partially capture abdominal adiposity, it does not integrate the synergistic effects of metabolic parameters. In recent years, novel obesity indices have emerged, including the lipid accumulation product (LAP), the triglyceride-glucose (TyG) index, and the Chinese visceral adiposity index (CVAI) (6). By integrating body composition and metabolic parameters in a multi-dimensional manner, these indices theoretically exhibit better alignment with the metabolic risk profiles of patients with PCOS.

However, most existing studies focus on only one or a few obesity indices, with study populations predominantly consisting of European and American (Caucasian) women—leaving a critical gap in systematic comparisons targeting Chinese women with PCOS (7). Notably, Chinese women differ from Western populations in key aspects: they are more prone to abdominal adiposity and have distinct metabolic baselines, including different normal ranges for fasting blood glucose (FBG) and triglycerides (TG) (8). Direct extrapolation of findings from foreign studies may thus lead to clinical misjudgment.

Based on the multicenter, large-sample data of the PCOSAct trial, this study systematically compared the predictive value of 7 traditional and novel obesity-related indices for IR and MetS in mainland Chinese women with PCOS: (1) traditional abdominal adiposity indices: waist-hip ratio (WHR) and waist-to-height ratio (WHtR); (2) lipid-integrated indices: LAP(integrating WC and TG) and visceral adiposity index (VAI) (integrating WC and TG/high-density lipoprotein cholesterol (HDL-C)); (3) glucose-integrated index: TyG index (reflects insulin sensitivity via TG and FBG); (4) population-optimized index: CVAI, established and optimized specifically for Chinese adults; (5) cardiometabolic index (CMI): linking adiposity to cardiovascular risk via anthropometric and lipid parameters. This study aimed to identify the optimal index with robust predictive efficacy, so as to provide a simple, practical tool for early screening of PCOS patients at high metabolic risk and to support individualized clinical intervention strategies.

## 2 Materials and methods

### 2.1 Participants

This secondary analysis utilized data from the PCOSAct trial, a multicenter study conducted in mainland China from 2011 to 2015. The trial protocol was approved by the Ethics Committee of the First Affiliated Hospital of Heilongjiang University of Chinese Medicine (Batch No. 2010HZYLL-010). This clinical trial was registered on chictr.org.cn (ChiCTR-TRC-12002081) and ClinicalTrials.gov (NCT01573858). Comprehensive descriptions of the trial design, eligibility criteria, and primary outcomes have been published elsewhere (9, 10).

A total of 1,000 women who met the *modified Rotterdam criteria* for PCOS were enrolled (11). Diagnosis required the presence of at least two of the following: (a) oligo- or anovulation; (b) clinical or biochemical hyperandrogenism (defined as a modified Ferriman–Gallwey score ≥ 5 for Chinese women) (12, 13); (c) polycystic ovarian morphology, defined as either ≥12 antral follicles (2–9 mm in diameter) or an ovarian volume ≥10 cm^3^. Individuals with conditions or medications that could confound the diagnosis or outcomes were excluded, including: (a) other causes of hyperandrogenism (congenital adrenal hyperplasia, Cushing’s syndrome, androgen-secreting tumors); (b) other endocrine disorders, including hyperprolactinemia, menopausal follicle-stimulating hormone (FSH) levels >15 mIU/mL, untreated thyroid dysfunction defined as thyroid-stimulating hormone (TSH) levels <0.2 mIU/mL or >5.5 mIU/mL, and poorly controlled type 1 or type 2 diabetes mellitus with glycated hemoglobin (HbA1c) > 7.0%; (c) significant hepatic or renal dysfunction, severe anemia, history of thromboembolic or cerebrovascular events, or other serious comorbidities; (d) use of hormonal medications (including oral contraceptives, depot progestogens, and Chinese herbal prescriptions) within the past 3 months, or any medications that could affect hormonal levels and ovarian function; (e) a history of pregnancy, abortion, delivery, or lactation within the previous 6 weeks.

### 2.2 Data collection

#### 2.2.1 Anthropometric measurements

During the baseline visit, anthropometric and hemodynamic parameters were recorded, including age, height, BMI (kg/m^2^), WC (cm), hip circumference (HC, cm), systolic and diastolic blood pressure (SBP and DBP, mmHg), and mean arterial pressure (MAP), the latter calculated as DBP + (SBP − DBP)/3 (14). Cutaneous manifestations of hyperandrogenism were evaluated using the modified Ferriman–Gallwey scoring system for hirsutism (15). This standardized scale evaluates excessive terminal hair growth in nine anatomical regions, including the face, trunk and extremities. Each region is scored from 0 to 4 points according to hair density, and a total score ≥8 is defined as clinical hirsutism. Additionally, a standardized counting method was applied to assess acne severity [None (0 score); Slight (1 score, less than 10 bumps (≥2 mm) on the face or trunk); Mild (2 scores, 10–20 bumps); Moderate (3 scores, over 20 bumps or fewer than 20 pustules); Severe (4 scores, over 20 pustules); Cystic acne (5 scores, inflammatory skin lesions ≥5 mm)]. Acanthosis nigricans (AN) was assessed clinically, which is characterized by hyperpigmented and thickened skin folds predominantly in the neck [None (1 score); Fine verrucous plaques with or without pigmentation in the neck or axillae (2 scores); Coarse verrucous plaque with or without pigmentation in the neck or axillae (3 scores); Coarse verrucous plaque with or without pigmentation on the neck, axillae, trunk and one pair of extremities (4 scores)].

#### 2.2.2 Laboratory parameters

Venous blood samples were obtained after a 12-h overnight fast on the third day of the menstrual cycle during baseline assessments. All biochemical measurements were conducted at the core laboratory of Heilongjiang University of Chinese Medicine. The analyzed parameters encompassed the following categories: (1) Glucose and insulin metabolism: fasting blood glucose (FBG, mmol/L; hexokinase method, Maker Biotechnology, China) and fasting insulin (FINS, pmol/L; electrochemiluminescence immunoassay, Roche Diagnostics, Switzerland); (2) Lipid profiles: high-density lipoprotein (HDL, mmol/L) and low-density lipoprotein (LDL, mmol/L; direct assays), triglycerides (TG, mmol/L) and total cholesterol (TC, mmol/L; enzymatic colorimetry, Wako Diagnostics, Japan), apolipoprotein A1 (APOA1, g/L), and apolipoprotein B (APOB, g/L; polyethylene glycol-enhanced immunoturbidimetry, Maker Biotechnology, China), lipoprotein (LP, mg/L; direct assays); (3) Reproductive hormones: progesterone (P, nmol/L), estradiol (E2, pmol/L), total testosterone (TT, nmol/L), sex hormone-binding globulin (SHBG, nmol/L), luteinizing hormone (LH, mIU/mL), follicle-stimulating hormone (FSH, mIU/mL), and anti-Müllerian hormone (AMH, ng/mL; chemiluminescent immunoassays, Siemens Healthineers, Germany), free testosterone (FT, pg./mL; radioimmunoassay); (4) Hepatic function indicators: alanine aminotransferase (ALT, U/L) and aspartate aminotransferase (AST, U/L; International Federation of Clinical Chemistry and Laboratory Medicine [IFCC] method), total bilirubin (TBIL, μmol/L), direct bilirubin (DBIL, μmol/L) and indirect bilirubin (IBIL, μmol/L; vanadic acid oxidation method), and total bile acid (TBA, μmol/L; enzyme cycling method); and (5) Renal and cardiac markers: blood urea nitrogen (UREA, mmol/L), creatinine (CREA, μmol/L; sarcosine oxidase method), beta 2-microglobulin (B2-MG, mg/L; immunoturbidimetry), cystatin C (CYSC, mg/L; enzyme-linked immunosorbent assay, ELISA), lactate dehydrogenase (LDH, U/L) and creatine kinase (CK, U/L; IFCC method), creatine kinase MB isoenzymes (CK-MB, U/L; selective inhibition method), and homocysteine (HCY, μmol/L; ELISA). IR index was assessed by the homeostasis model assessment (HOMA-IR), calculated as [FINS (pmol/L)/6] × FBG (mmol/L)/22.5, where 1 mIU/mL insulin = 6 pmol/L. Insulin sensitivity was additionally evaluated using the quantitative insulin sensitivity check index (QUICKI), defined as 1/[ln(FINS (pmol/L)/6) + ln(FBG (mmol/L) × 18)], where FINS (pmol/L)/6 converts to μU/mL, FBG (mmol/L) × 18 converts to mg/dL, and ln denotes the natural logarithm. The free androgen index (FAI) was calculated as (TT (nmol/L)/SHBG (nmol/L)) × 100.

The following indices were derived using established formulas: WHR was determined as WC (cm) divided by HC (cm) (16); WHtR as WC (cm) divided by height (cm) (17); VAI for women was calculated as [WC (cm)/(36.58 + 1.89 × BMI (kg/m^2^))] × [TG (mmol/L)/0.81] × [1.52/HDL-C (mmol/L)] (18); CVAI was calculated as −187.32 + 1.71 × age (years) + 4.23 × BMI (kg/m^2^) + 1.12 × WC (cm) + 39.76 × ln[TG (mmol/L)] − 11.66 × HDL-C (mmol/L), where ln denotes the natural logarithm (19); LAP as (WC (cm) − 58) × TG (mmol/L) (20); TyG index was calculated as ln[(TG (mmol/L) × 88.57) × (FBG (mmol/L) × 18)/2], where TG (mmol/L) × 88.57 converts to mg/dL and FBG (mmol/L) × 18 converts to mg/dL (21); and the CMI index was calculated as the WC (cm)/height (cm) × TG (mmol/L)/HDL-C (mmol/L) (22).

Additionally, HOMA-IR value ≥2.69 was used to define IR (23). Metabolic syndrome (MetS) was diagnosed based on Chinese female-specific criteria, which require the presence of at least three of the following components: WC ≥ 85 cm; SBP ≥ 130 mmHg and/or DBP ≥ 85 mmHg; TG ≥ 1.7 mmol/L; HDL-C < 1.04 mmol/L; and FBG ≥ 6.1 mmol/L (24).

### 2.3 Statistical analyses

Statistical analyses were conducted using IBM SPSS Statistics (version 26.0). Normality of continuous variables was evaluated using the Shapiro–Wilk test. Normally distributed continuous variables are expressed as mean ± standard deviation (SD), while non-normally distributed variables are presented as median (interquartile range) [M (P25, P75)]. Group comparisons (IR− vs. IR + or MetS− vs. MetS+) were performed with the Student’s t-test for normally distributed data and the Mann–Whitney *U* test for non-normally distributed data. The associations between body composition indices and metabolic parameters—including WC, SBP, DBP, HDL-C, TG, FBG, and FINS—were examined using Pearson’s correlation for normally distributed variables and Spearman’s correlation for non-normally distributed variables.

The discriminatory power of WHR, WHtR, VAI, CVAI, LAP, TyG, and CMI for identifying IR and MetS was evaluated using receiver operating characteristic (ROC) curve analysis, with the area under the curve (AUC) serving as a measure of predictive accuracy. Optimal cutoff points for these indices were established by maximizing Youden’s J statistic (sensitivity + specificity − 1). A *p*-value below 0.05 was regarded as statistically significant.

## 3 Result

**Figure 1:**
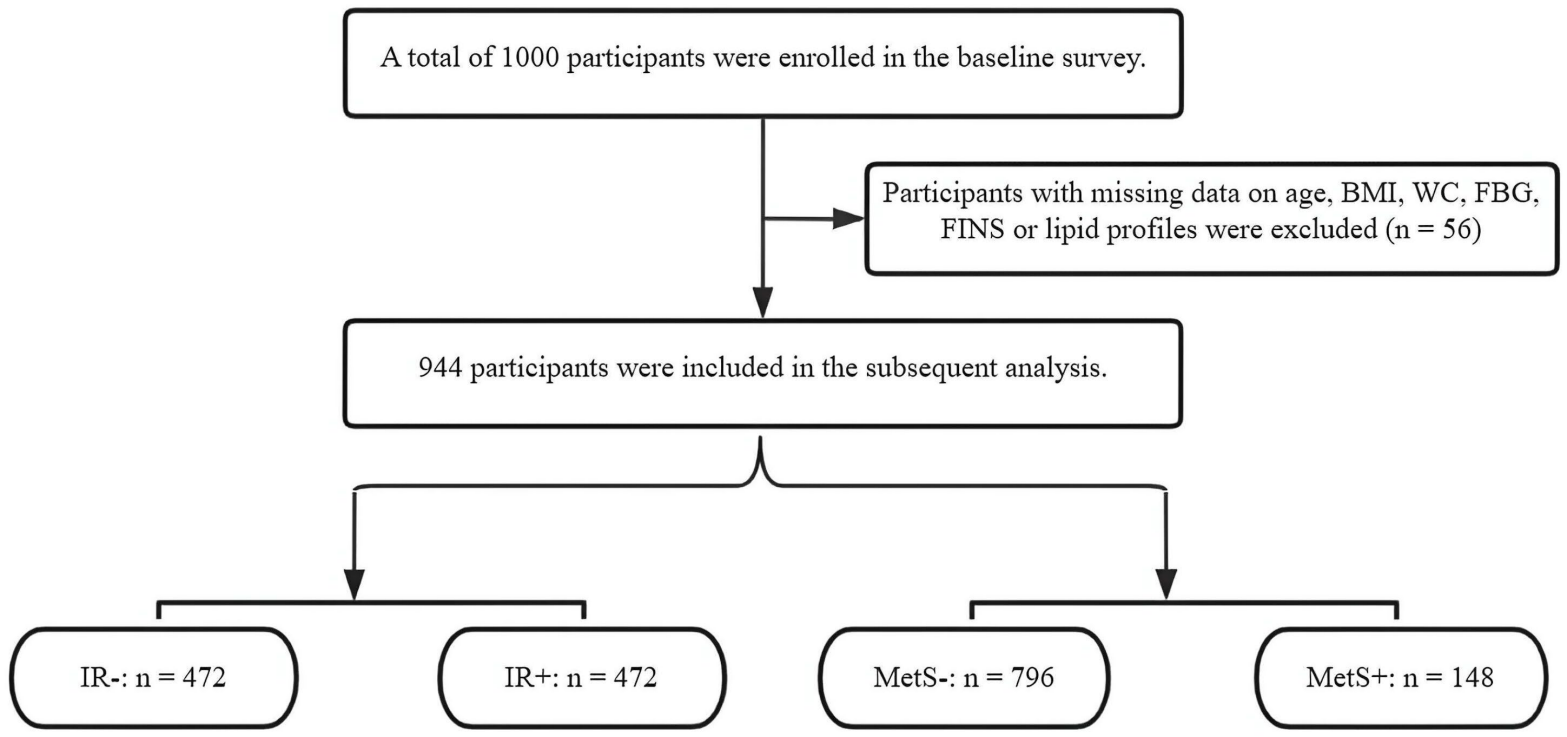
Flowchart of the study population.

Of the 1,000 enrolled PCOS patients in this clinical trial, 56 were excluded due to missing baseline measurements of age, BMI, WC, FBG, FINS, or lipid profiles. Consequently, 944 patients were included in the subsequent secondary analysis (Figure 1).

### 3.1 The anthropometric, biochemical characteristics of patients with IR or without IR

As shown in Table 1, IR was present in 472 patients (50%). Compared to non-IR PCOS patients, those with IR demonstrated significantly elevated anthropometric measures, including height, weight, BMI, WC, HC, SBP, DBP, MAP, and AN scores (*p* < 0.001). Regarding glucolipid metabolism, the IR group exhibited higher levels of FBG, FINS, HOMA-IR, LDL, TG, TC, APOB, and the APOB/APOA1 ratio, alongside lower QUICKI and HDL levels (all *p* < 0.001). Significant differences were also observed in reproductive hormones, with higher FT and FAI but lower E2, SHBG, LH, LH/FSH ratio, and AMH in the IR group (all *p* < 0.05). In terms of hepatic/renal and cardiac markers, ALT was increased, whereas TBIL, DBIL, IBIL, CREA, and CK-MB were decreased in IR patients compared to non-IR counterparts (all *p* < 0.01).

**Table 1:** Descriptive statistics for all subjects, IR−, and IR+.

Variables	All	IR− (*n* = 472)	IR+ (*n* = 472)	*p* value
Anthropometric parameters				
Age, year, mean (SD)	27.92 (3.31)	27.9 (3.23)	27.93 (3.39)	0.831
Height, cm, mean (SD)	161.19 (5.09)	160.61 (4.97)	161.77 (5.14)	<0.001
Weight, kg, mean (SD)	63.01 (12.42)	57.1 (9.28)	68.93 (12.35)	<0.001
BMI, kg/m^2^, mean (SD)	24.19 (4.26)	22.12 (3.32)	26.26 (4.09)	<0.001
WC, cm, mean (SD)	85.41 (11.52)	80.04 (9.27)	90.78 (11.05)	<0.001
HC, cm, mean (SD)	98.39 (8.56)	94.52 (7.04)	102.26 (8.19)	<0.001
SBP, mmHg, mean (SD)	112.36 (9.31)	110.87 (9.99)	113.86 (8.32)	<0.001
DBP, mmHg, mean (SD)	74.9 (7.82)	74.08 (8.24)	75.71 (7.31)	0.001
MAP, mmHg, mean (SD)	87.38 (7.56)	86.34 (8.09)	88.43 (6.85)	<0.001
Hirsutism score, mean (SD)	3.01 (2.77)	2.92 (2.63)	3.1 (2.9)	0.572
Acne score, mean (SD)	0.44 (0.77)	0.43 (0.77)	0.44 (0.76)	0.521
AN score, mean (SD)	1.21 (0.48)	1.13 (0.37)	1.29 (0.55)	<0.001
Glucose and insulin metabolism				
FBG, mmol/L, mean (SD)	5.04 (0.96)	4.64 (0.86)	5.43 (0.89)	<0.001
FINS, pmol/L, mean (SD)	94.82 (84.08)	46.57 (19.1)	143.08 (95.52)	<0.001
HOMA-IR, mean (SD)	3.76 (4.09)	1.6 (0.67)	5.93 (4.86)	<0.001
QUICKI, mean (SD)	0.33 (0.04)	0.37 (0.04)	0.3 (0.02)	<0.001
Lipid profiles				
HDL, mmol/L, mean (SD)	1.28 (0.37)	1.33 (0.38)	1.22 (0.35)	<0.001
LDL, mmol/L, mean (SD)	2.98 (0.87)	2.81 (0.77)	3.14 (0.94)	<0.001
TG, mmol/L, mean (SD)	1.57 (0.91)	1.21 (0.62)	1.94 (1)	<0.001
TC, mmol/L, mean (SD)	4.74 (1.08)	4.53 (0.95)	4.96 (1.16)	<0.001
APOA1, g/L, mean (SD)	1.51 (0.32)	1.51 (0.32)	1.51 (0.31)	0.821
APOB, g/L, mean (SD)	0.9 (0.29)	0.8 (0.23)	1 (0.31)	<0.001
APOB/APOA1 ratio, mean (SD)	0.61 (0.2)	0.55 (0.17)	0.68 (0.21)	<0.001
LP, mg/L, mean (SD)	129.69 (98.59)	130.57 (102.34)	128.81 (94.8)	0.697
Reproductive hormones				
P, nmol/L, mean (SD)	2.54 (4.8)	2.66 (5.51)	2.42 (3.96)	0.332
E2, pmol/L, mean (SD)	269.11 (317.34)	281.33 (309.25)	256.92 (325.09)	0.048
TT, nmol/L, mean (SD)	1.67 (0.65)	1.64 (0.62)	1.69 (0.68)	0.286
FT, pg./ml, mean (SD)	2.29 (0.84)	2.23 (0.87)	2.35 (0.81)	0.005
SHBG, nmol/L, mean (SD)	42.66 (30.37)	51.85 (30.58)	33.52 (27.27)	<0.001
FAI, mean (SD)	5.84 (4.41)	4.47 (3.53)	7.2 (4.77)	<0.001
LH, mIU/mL, mean (SD)	10.53 (5.95)	11.58 (6.43)	9.48 (5.23)	<0.001
FSH, mIU/mL, mean (SD)	6.1 (1.67)	6.22 (1.64)	5.97 (1.69)	0.013
LH/FSH ratio, mean (SD)	1.79 (1.14)	1.92 (1.08)	1.65 (1.18)	<0.001
AMH, ng/mL, mean (SD)	12.08 (6.38)	13.05 (6.71)	11.11 (5.89)	<0.001
Hepatic function parameters				
ALT, U/L, mean (SD)	9 (8.63)	7.63 (6.61)	10.38 (10.08)	<0.001
AST, U/L, mean (SD)	13.06 (7.36)	12.61 (6.41)	13.52 (8.2)	0.248
TBIL, μmol/L, mean (SD)	6.39 (3.16)	7.1 (3.41)	5.68 (2.71)	<0.001
DBIL, μmol/L, mean (SD)	2.3 (1.42)	2.62 (1.5)	1.98 (1.27)	<0.001
IBIL, μmol/L, mean (SD)	4.14 (2.06)	4.52 (2.17)	3.76 (1.87)	<0.001
TBA, μmol/L, mean (SD)	1.97 (2.97)	2.09 (3.62)	1.85 (2.11)	0.165
Renal and cardiac markers				
UREA, mmol/L, mean (SD)	4.38 (1.28)	4.37 (1.37)	4.39 (1.18)	0.523
CREA, μmol/L, mean (SD)	42.98 (10.76)	43.9 (10.67)	42.05 (10.77)	0.005
B2-MG, mg/L, mean (SD)	1.31 (0.4)	1.32 (0.42)	1.3 (0.37)	0.651
CYSC, mg/L, mean (SD)	0.46 (0.15)	0.46 (0.15)	0.47 (0.15)	0.726
HCY, μmol/L, mean (SD)	8.34 (4.86)	8.29 (4.69)	8.39 (5.03)	0.999
LDH, U/L, mean (SD)	87.7 (44.8)	88.46 (44.36)	86.94 (45.28)	0.597
CK, U/L, mean (SD)	55.62 (31.77)	55.36 (29.52)	55.88 (33.9)	0.648
CK-MB, U/L, mean (SD)	2.91 (5.49)	3.36 (6.36)	2.28 (3.85)	<0.001

Statistical methods: Student’s *t*-test. BMI, body mass index; WC, waist circumference; HC, hip circumference; SBP, systolic blood pressure; DBP, diastolic blood pressure; MAP, mean arterial pressure; AN, acanthosis nigricans; FBG, fasting blood glucose; FINS, fasting insulin; HOMA-IR, homeostatic model assessment-insulin resistance; QUICKI, quantitative insulin sensitivity check index; HDL, high-density lipoprotein; LDL, low-density lipoprotein; TG, triglycerides; TC, total cholesterol; ApoA1, apolipoprotein A1; ApoB, apolipoprotein B; LP, lipoprotein; P, progesterone; E2, estradiol; TT, total testosterone; FT, free testosterone; SHBG, sex hormone-binding globulin; FAI, free androgen index; LH, luteinizing hormone; FSH, follicle-stimulating hormone; AMH, anti-Müllerian hormone; ALT, alanine aminotransferase; AST, aspartate aminotransferase; TBIL, total bilirubin; DBIL, direct bilirubin; IBIL, indirect bilirubin; TBA, total bile acid; UREA, blood urea nitrogen; CREA, creatinine; B2-MG, beta 2-microglobulin; CYSC, cystatin C; HCY, homocysteine; LDH, lactate dehydrogenase; CK, creatine kinase; CK-MB, creatine kinase MB isoenzymes.

### 3.2 The anthropometric, biochemical characteristics of patients with MetS or without MetS

As presented in Table 2, MetS was identified in 148 patients (15.68%). Compared to PCOS patients without MetS, those with MetS were older and exhibited significantly greater anthropometric measures including height, weight, BMI, WC, HC, SBP, DBP, MAP, hirsutism scores, and AN scores (all *p* < 0.05). Notable disparities were also observed in glucolipid metabolism parameters. The MetS group displayed higher levels of FBG, FINS, HOMA-IR, LDL, TG, TC, ApoB, and ApoB/ApoA1 ratio, along with lower QUICKI, HDL, and APOA1 levels (all *p* < 0.01). In terms of reproductive hormone profiles, the MetS group had elevated FT and FAI values, but lower SHBG, LH, FSH, LH/FSH ratio, and AMH levels (all *p* < 0.05). Additionally, significant differences emerged in hepatic/renal function markers and myocardial enzymes, with the MetS group showing increased ALT, AST, CYSC, HCY and B2-MG, alongside decreased DBIL (all *p* < 0.01).

**Table 2:** Descriptive statistics for all subjects, MetS−, and MetS+.

Variables	All	MetS− (*n* = 796)	MetS+ (*n* = 148)	*p* value
Anthropometric parameters				
Age, year, mean (SD)	27.92 (3.31)	27.77 (3.24)	28.72 (3.54)	0.002
Height, cm, mean (SD)	161.19 (5.09)	160.96 (5.04)	162.44 (5.2)	0.001
Weight, kg, mean (SD)	63.01 (12.42)	60.97 (11.49)	74.01 (11.47)	<0.001
BMI, kg/m^2^, mean (SD)	24.19 (4.26)	23.48 (3.98)	27.99 (3.69)	<0.001
WC, cm, mean (SD)	85.41 (11.52)	83.46 (10.78)	95.89 (9.59)	<0.001
HC, cm, mean (SD)	98.39 (8.56)	97.19 (8.18)	104.81 (7.66)	<0.001
SBP, mmHg, mean (SD)	112.36 (9.31)	111.4 (9.16)	117.53 (8.42)	<0.001
DBP, mmHg, mean (SD)	74.9 (7.82)	73.95 (7.51)	79.98 (7.54)	<0.001
MAP, mmHg, mean (SD)	87.38 (7.56)	86.43 (7.3)	92.49 (6.92)	<0.001
Hirsutism score, mean (SD)	3.01 (2.77)	2.92 (2.74)	3.45 (2.85)	0.024
Acne score, mean (SD)	0.44 (0.77)	0.45 (0.77)	0.37 (0.77)	0.136
AN score, mean (SD)	1.21 (0.48)	1.18 (0.46)	1.32 (0.55)	<0.001
Glucose and insulin metabolism				
FBG, mmol/L, mean (SD)	5.04 (0.96)	4.94 (0.83)	5.55 (1.36)	<0.001
FINS, pmol/L, mean (SD)	94.82 (84.08)	83.57 (73.5)	155.35 (108.46)	<0.001
HOMA-IR, mean (SD)	3.76 (4.09)	3.19 (3.28)	6.81 (6.16)	<0.001
QUICKI, mean (SD)	0.33 (0.04)	0.34 (0.04)	0.3 (0.03)	<0.001
Lipid profiles				
HDL, mmol/L, mean (SD)	1.28 (0.37)	1.33 (0.35)	1 (0.35)	<0.001
LDL, mmol/L, mean (SD)	2.98 (0.87)	2.92 (0.81)	3.28 (1.1)	<0.001
TG, mmol/L, mean (SD)	1.57 (0.91)	1.39 (0.78)	2.55 (0.93)	<0.001
TC, mmol/L, mean (SD)	4.74 (1.08)	4.68 (1.01)	5.09 (1.36)	0.002
APOA1, g/L, mean (SD)	1.51 (0.32)	1.53 (0.31)	1.36 (0.29)	<0.001
APOB, g/L, mean (SD)	0.9 (0.29)	0.86 (0.26)	1.11 (0.31)	<0.001
APOB/APOA1 ratio, mean (SD)	0.61 (0.2)	0.57 (0.18)	0.82 (0.19)	<0.001
LP, mg/L, mean (SD)	129.69 (98.59)	127.42 (95.37)	141.9 (113.92)	0.119
Reproductive hormones				
P, nmol/L, mean (SD)	2.54 (4.8)	2.55 (4.92)	2.49 (4.08)	0.286
E2, pmol/L, mean (SD)	269.11 (317.34)	270.06 (322.62)	264.03 (288.33)	0.694
TT, nmol/L, mean (SD)	1.67 (0.65)	1.66 (0.64)	1.71 (0.71)	0.391
FT, pg./ml, mean (SD)	2.29 (0.84)	2.25 (0.84)	2.5 (0.8)	<0.001
SHBG, nmol/L, mean (SD)	42.66 (30.37)	45.6 (31.23)	27.04 (18.77)	<0.001
FAI, mean (SD)	5.84 (4.41)	5.39 (4.18)	8.25 (4.86)	<0.001
LH, mIU/mL, mean (SD)	10.53 (5.95)	10.89 (6.1)	8.55 (4.6)	<0.001
FSH, mIU/mL, mean (SD)	6.1 (1.67)	6.15 (1.69)	5.8 (1.52)	0.013
LH/FSH ratio, mean (SD)	1.79 (1.14)	1.84 (1.19)	1.49 (0.75)	0.001
AMH, ng/mL, mean (SD)	12.08 (6.38)	12.45 (6.47)	10.11 (5.52)	<0.001
Hepatic function parameters				
ALT, U/L, mean (SD)	9 (8.63)	8.27 (7.73)	13.01 (11.67)	<0.001
AST, U/L, mean (SD)	13.06 (7.36)	12.49 (6.63)	16.19 (9.98)	<0.001
TBIL, μmol/L, mean (SD)	6.39 (3.16)	6.47 (3.21)	5.93 (2.85)	0.053
DBIL, μmol/L, mean (SD)	2.3 (1.42)	2.37 (1.41)	1.91 (1.44)	<0.001
IBIL, μmol/L, mean (SD)	4.14 (2.06)	4.15 (2.05)	4.1 (2.11)	0.826
TBA, μmol/L, mean (SD)	1.97 (2.97)	2.03 (3.15)	1.62 (1.57)	0.12
Renal and cardiac markers				
UREA, mmol/L, mean (SD)	4.38 (1.28)	4.36 (1.3)	4.49 (1.15)	0.206
CREA, μmol/L, mean (SD)	42.98 (10.76)	42.9 (10.7)	43.41 (11.09)	0.539
B2-MG, mg/L, mean (SD)	1.31 (0.4)	1.29 (0.39)	1.41 (0.42)	<0.001
CYSC, mg/L, mean (SD)	0.46 (0.15)	0.46 (0.15)	0.51 (0.15)	<0.001
HCY, μmol/L, mean (SD)	8.34 (4.86)	8.27 (5.01)	8.7 (3.94)	0.034
LDH, U/L, mean (SD)	87.7 (44.8)	86.39 (43.3)	94.76 (51.8)	0.09
CK, U/L, mean (SD)	55.62 (31.77)	55.55 (32.9)	56.03 (24.81)	0.432
CK-MB, U/L, mean (SD)	2.91 (5.49)	2.95 (5.67)	2.58 (3.53)	0.482

Statistical methods: Student’s *t*-test. BMI, body mass index; WC, waist circumference; HC, hip circumference; SBP, systolic blood pressure; DBP, diastolic blood pressure; MAP, mean arterial pressure; AN, acanthosis nigricans; FBG, fasting blood glucose; FINS, fasting insulin; HOMA-IR, homeostatic model assessment-insulin resistance; QUICKI, quantitative insulin sensitivity check index; HDL, high-density lipoprotein; LDL, low-density lipoprotein; TG, triglycerides; TC, total cholesterol; ApoA1, apolipoprotein A1; ApoB, apolipoprotein B; LP, lipoprotein; P, progesterone; E2, estradiol; TT, total testosterone; FT, free testosterone; SHBG, sex hormone-binding globulin; FAI, free androgen index; LH, luteinizing hormone; FSH, follicle-stimulating hormone; AMH, anti-Müllerian hormone; ALT, alanine aminotransferase; AST, aspartate aminotransferase; TBIL, total bilirubin; DBIL, direct bilirubin; IBIL, indirect bilirubin; TBA, total bile acid; UREA, blood urea nitrogen; CREA, creatinine; B2-MG, beta 2-microglobulin; CYSC, cystatin C; HCY, homocysteine; LDH, lactate dehydrogenase; CK, creatine kinase; CK-MB, creatine kinase MB isoenzymes.

### 3.3 The correlations between various indices and metabolic parameters

As summarized in Table 3. Strong correlations were observed for WHR, WHtR, VAI, CVAI, LAP, TyG, and CMI with WC, SBP, DBP, HDL, TG, FBG, and FINS (all *p* < 0.001). Among these indices, WHR correlated most strongly with WC (*r* = 0.799), followed by FINS (*r* = 0.390). WHtR showed the highest correlation with WC (*r* = 0.968), then with FINS (*r* = 0.539). VAI demonstrated the strongest association with TG (*r* = 0.912), followed by an inverse correlation with HDL (*r* = −0.678) and a positive correlation with FINS (*r* = 0.509). CVAI was most strongly correlated with WC (*r* = 0.871), and then with FINS (*r* = 0.637) and TG (*r* = 0.633). LAP exhibited the highest correlation with TG (*r* = 0.860), followed by WC (*r* = 0.776) and FINS (*r* = 0.628). Similarly, the TyG index showed the strongest association with TG (*r* = 0.951), followed by FINS (*r* = 0.551) and FBG (*r* = 0.482). Finally, CMI correlated most strongly with TG (*r* = 0.633), followed by FINS (*r* = 0.550) and WC (*r* = 0.539).

**Table 3:** Bivariate correlation coefficients between indices of adiposity and metabolic characteristics.

Variables	WHR	WHtR	VAI	CVAI	LAP	TyG	CMI
WC (cm)	0.799^***^	0.968^***^	0.471^***^	0.871^***^	0.776^***^	0.385^***^	0.539^***^
SBP (mmHg)	0.245^***^	0.289^***^	0.202^***^	0.335^***^	0.285^***^	0.188^***^	0.228^***^
DBP (mmHg)	0.222^***^	0.23^***^	0.14^***^	0.231^***^	0.218^***^	0.155^***^	0.156^***^
HDL (mmol/L)	−0.218^***^	−0.27^***^	−0.678^***^	−0.465^***^	−0.394^***^	−0.263^***^	−0.67^***^
TG (mmol/L)	0.296^***^	0.375^***^	0.912^***^	0.633^***^	0.86^***^	0.951^***^	0.906^***^
FBG (mmol/L)	0.161^***^	0.22^***^	0.111^***^	0.214^***^	0.269^***^	0.482^***^	0.13^***^
FINS (pmol/L)	0.39^***^	0.539^***^	0.509^***^	0.637^***^	0.628^***^	0.551^***^	0.55^***^

*p < 0.05; **p < 0.01; ***p < 0.001. Statistical methods: Pearson’s correlation/Spearman’s correlation. WC, waist circumference; SBP, systolic blood pressure; DBP, diastolic blood pressure; HDL, high-density lipoprotein; TG, triglycerides; FBG, fasting blood glucose; FINS, fasting insulin; WHR, waist-to-hip ratio; WHtR, waist-to-height ratio; VAI, visceral adiposity index; CVAI, Chinese visceral adiposity index; LAP, lipid accumulation product; TyG, triglyceride-glucose index; CMI, cardiometabolic index.

### 3.4 The correlations between various indices and biochemical parameters

As shown in Table 4, overall, significant and widespread correlations were observed between various adiposity indices and multiple biochemical parameters encompassing sex hormones, liver function, renal function, and myocardial enzymes.

**Table 4:** Bivariate correlation coefficients between indices of adiposity and biochemical parameters.

Variables	WHR	WHtR	VAI	CVAI	LAP	TyG	CMI
P (nmol/L)	−0.045	−0.066^*^	−0.143^***^	−0.128^***^	−0.108^**^	−0.082^*^	−0.143^***^
E2 (pmol/L)	−0.074^*^	−0.104^**^	−0.113^***^	−0.151^***^	−0.124^***^	−0.056	−0.122^***^
TT (nmol/L)	0.051	0.039	−0.048	0.013	0.005	−0.007	−0.036
FT (pg/ml)	0.116^***^	0.144^***^	0.109^**^	0.184^***^	0.127^***^	0.032	0.129^***^
SHBG (nmol/L)	−0.384^***^	−0.466^***^	−0.433^***^	−0.542^***^	−0.482^***^	−0.325^***^	−0.471^***^
FAI	0.342^***^	0.403^***^	0.334^***^	0.452^***^	0.395^***^	0.258^***^	0.372^***^
LH (mIU/mL)	−0.149^***^	−0.238^***^	−0.196^***^	−0.29^***^	−0.214^***^	−0.094^**^	−0.214^***^
FSH (mIU/mL)	−0.068^*^	−0.084^*^	−0.129^***^	−0.122^***^	−0.083^*^	−0.025	−0.129^***^
LH/FSH ratio	−0.141^***^	−0.224^***^	−0.137^***^	−0.257^***^	−0.184^***^	−0.079^*^	−0.159^***^
AMH (ng/mL)	−0.107^**^	−0.132^***^	−0.173^***^	−0.202^***^	−0.18^***^	−0.134^***^	−0.18^***^
ALT (U/L)	0.283^***^	0.323^***^	0.337^***^	0.384^***^	0.384^***^	0.283^***^	0.361^***^
AST (U/L)	0.204^***^	0.198^***^	0.175^***^	0.212^***^	0.202^***^	0.105^**^	0.193^***^
TBIL (μmol/L)	−0.069^*^	−0.178^***^	−0.151^***^	−0.183^***^	−0.217^***^	−0.206^***^	−0.167^***^
DBIL (μmol/L)	−0.077^*^	−0.183^***^	−0.21^***^	−0.214^***^	−0.263^***^	−0.242^***^	−0.223^***^
IBIL (μmol/L)	−0.044	−0.145^***^	−0.062	−0.133^***^	−0.129^***^	−0.121^***^	−0.079^*^
TBA (μmol/L)	−0.094^**^	−0.123^***^	−0.111^**^	−0.168^***^	−0.136^***^	−0.09^**^	−0.12^***^
UREA (mmol/L)	0.08^*^	0.095^**^	−0.018	0.065^*^	−0.003	−0.065^*^	0.001
CREA (μmol/L)	−0.014	−0.021	−0.033	−0.017	−0.087^**^	−0.134^***^	−0.033
B2-MG (mg/L)	0.07^*^	0.122^***^	0.101^**^	0.136^***^	0.075^*^	−0.03	0.113^**^
CYSC (mg/L)	0.095^**^	0.126^***^	0.107^**^	0.151^***^	0.072^*^	−0.019	0.122^***^
HCY (μmol/L)	0.097^**^	0.12^***^	0.067^*^	0.128^***^	0.055	−0.081^*^	0.079^*^
LDH (U/L)	0.104^**^	0.111^**^	0.145^***^	0.131^***^	0.095^**^	0.013	0.151^***^
CK (U/L)	0.075^*^	0.095^**^	0.011	0.1^**^	0.028	−0.057	0.031
CK-MB (U/L)	−0.125^**^	−0.141^**^	−0.221^***^	−0.204^***^	−0.233^***^	−0.251^***^	−0.218^***^

*p < 0.05; **p < 0.01; ***p < 0.001. Statistical methods: Pearson’s correlation/Spearman’s correlation. P, progesterone; E2, estradiol; TT, total testosterone; FT, free testosterone; SHBG, sex hormone-binding globulin; FAI, free androgen index; LH, luteinizing hormone; FSH, follicle-stimulating hormone; AMH, anti-Müllerian hormone; ALT, alanine aminotransferase; AST, aspartate aminotransferase; TBIL, total bilirubin; DBIL, direct bilirubin; IBIL, indirect bilirubin; TBA, total bile acid; UREA, blood urea nitrogen; CREA, creatinine; B2-MG, beta 2-microglobulin; CYSC, cystatin C; HCY, homocysteine; LDH, lactate dehydrogenase; CK, creatine kinase; CK-MB, creatine kinase MB isoenzymes.

Regarding sex hormones, P showed a negative but non-significant correlation with WHR and negative and significant correlations with the other adiposity indices (all *p* < 0.05), with the strongest associations seen for the VAI and CMI (both *r* = −0.143). SHBG was negatively correlated with all indices (all *p* < 0.001), most strongly with the CVAI (*r* = −0.542). The FAI demonstrated positive correlations with all indices (all *p* < 0.001), also strongest with CVAI (*r* = 0.452). FT showed positive correlations with all indices except the TyG (all *p* < 0.01), while E2 was negatively correlated with most indices except TyG (all *p* < 0.05); CVAI showed the strongest correlations for both FT (*r* = 0.452) and E2 (*r* = −0.151). LH, FSH, the LH/FSH ratio, and AMH were predominantly negatively correlated with the indices (all *p* < 0.05). No significant correlations were found for TT.

For hepatic function markers, both ALT and AST were positively correlated with most indices (all *p* < 0.001). ALT correlated most strongly with CVAI and LAP (*r* = 0.384 for both), while AST correlated most strongly with CVAI (*r* = 0.212). Bilirubin fractions (TBIL, DBIL, and IBIL) and TBA were generally negatively correlated with the indices (all *p* < 0.05). TBIL and DBIL correlated most strongly with LAP (*r* = −0.217 and *r* = −0.263, respectively), whereas TBA correlated most strongly with CVAI (*r* = −0.168).

Among renal function markers, B2-MG and CYSC were positively correlated with most indices (all *p* < 0.05), except TyG, and both showed the strongest correlations with CVAI (*r* = 0.136 and *r* = 0.151, respectively). Conversely, CREA was negatively correlated with LAP and TyG (both *p* < 0.01), most strongly with TyG (*r* = −0.134).

For myocardial enzymes, LDH was positively correlated with all indices except TyG (all p < 0.01), most strongly with CMI (*r* = 0.151). CK showed positive correlations only with WHR, WHtR, and CVAI (all *p* < 0.05), strongest with CVAI (*r* = 0.1). In contrast, CK-MB was generally negatively correlated (all *p* < 0.01), most strongly with TyG (*r* = −0.251).

### 3.5 Diagnostic performance of various indices for IR and MetS

**Figure 2:**
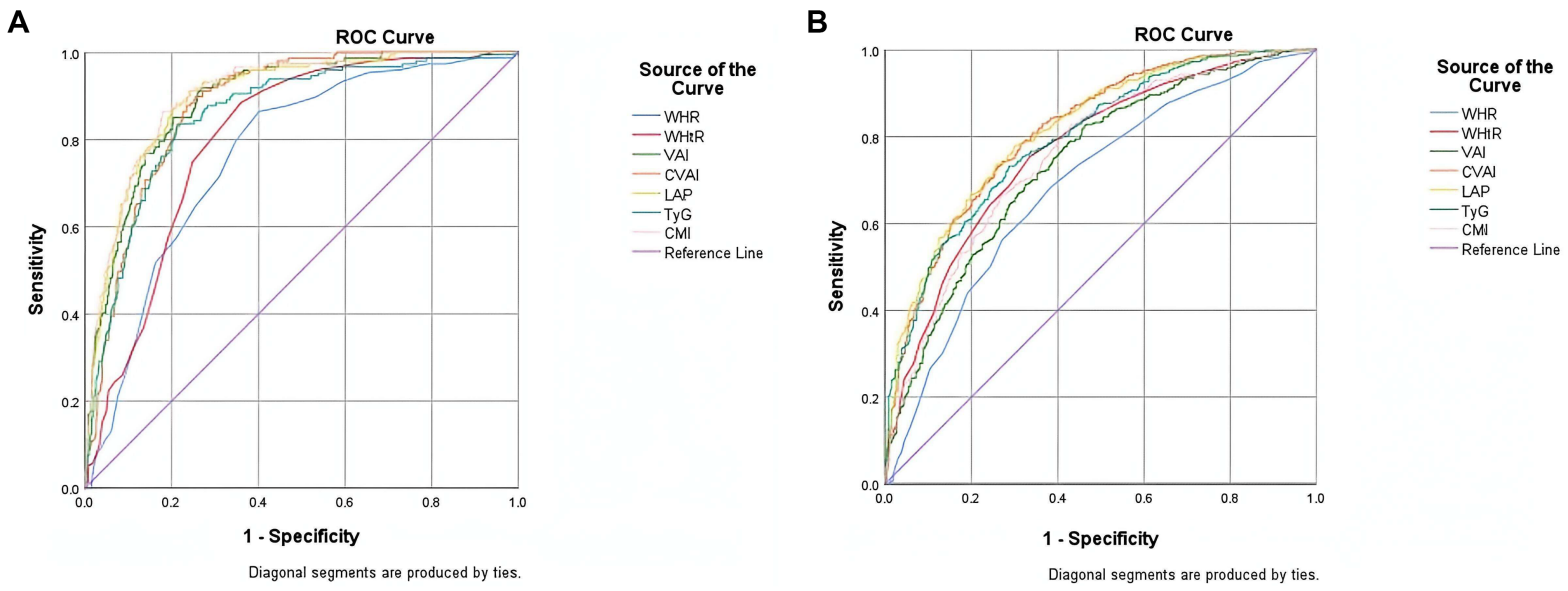
The results of ROC curve analysis regarding the predictability of indices of adiposity in IR **(A)** and MetS **(B)**.

As presented in Figure 2 and Table 5, all evaluated adiposity indices demonstrated statistically significant discriminative ability, with area under the AUC values significantly greater than 0.5 (all *p* < 0.001), supporting their potential role in predicting metabolic abnormalities.

**Table 5:** The predictive value of indices of adiposity in detecting IR and MetS.

Predictors	AUC (95% CI)	*p*-value	Cut-off value	Sensitivity	Specificity	Youden index
IR						
WHR	0.689 (0.655–0.723)	<0.001	0.86	68.20%	61.90%	0.301
WHtR	0.768 (0.738–0.797)	<0.001	0.52	75.40%	66.50%	0.419
VAI	0.740 (0.709–0.771)	<0.001	2.07	69.00%	67.60%	0.366
CVAI	0.813 (0.786–0.840)	<0.001	45.41	74.30%	72.90%	0.472
LAP	0.815 (0.789–0.842)	<0.001	32.15	78.00%	69.90%	0.479
TyG	0.796 (0.768–0.824)	<0.001	8.53	73.30%	71.20%	0.445
CMI	0.761 (0.730–0.791)	<0.001	0.43	82.80%	56.80%	0.396
MetS						
WHR	0.767 (0.729–0.804)	<0.001	0.87	86.50%	59.90%	0.464
WHtR	0.803 (0.770–0.835)	<0.001	0.54	88.50%	64.10%	0.526
VAI	0.893 (0.868–0.917)	<0.001	2.76	91.20%	73.90%	0.651
CVAI	0.881 (0.857–0.904)	<0.001	61.08	89.80%	74.60%	0.644
LAP	0.898 (0.874–0.923)	<0.001	48.35	91.20%	76.00%	0.672
TyG	0.858 (0.826–0.890)	<0.001	8.90	83.10%	78.90%	0.62
CMI	0.905 (0.882–0.928)	<0.001	0.94	86.40%	82.20%	0.686

Statistical methods: Receiver operating characteristic curve analysis. WHR, waist-to-hip ratio; WHtR, waist-to-height ratio; VAI, visceral adiposity index; CVAI, Chinese visceral adiposity index; LAP, lipid accumulation product; TyG, triglyceride-glucose index; CMI, cardiometabolic index; IR, insulin resistance; MetS, metabolic syndrome.

For the identification of IR, the LAP and CVAI showed the highest diagnostic performance, with AUCs of 0.815 and 0.813, respectively. At a cut-off value of 32.15, LAP provided a sensitivity of 78.0% and a specificity of 69.9% (Youden’s index = 0.479). CVAI, using a threshold of 45.41, achieved a sensitivity of 74.3% and a specificity of 72.9% (Youden’s index = 0.472). The TyG index also exhibited strong predictive capacity, with an AUC of 0.796. At the optimal cut-off of 8.53, TyG yielded a sensitivity of 73.3% and a specificity of 71.2% (Youden’s index = 0.445).

In predicting MetS, the CMI, LAP, and VAI displayed the highest diagnostic accuracy, with AUCs of 0.905, 0.898, and 0.893, respectively. A CMI cut-off of 0.94 corresponded to a sensitivity of 86.4% and a specificity of 82.2% (Youden’s index = 0.686). LAP, at a cut-off of 48.35, showed a sensitivity of 91.2% and a specificity of 76.0% (Youden’s index = 0.672), while VAI at a threshold of 2.76 provided a sensitivity of 91.2% and a specificity of 73.9% (Youden’s index = 0.651).

## 4 Discussion

The present study represents one of the most comprehensive evaluations to date, systematically comparing the predictive capacities of seven distinct adiposity indices—encompassing both traditional (WHR, WHtR) and novel (VAI, CVAI, LAP, TyG, CMI) metrics—for identifying IR and MetS in a large, well-characterized cohort of Chinese women with PCOS. Our findings suggest that these indices are not only strongly correlated with a wide spectrum of metabolic and biochemical parameters but also may exhibit significant and often superior discriminatory power for detecting these prevalent and interconnected metabolic complications. Crucially, the performance of these indices varied in a condition-specific manner, highlighting the distinct pathophysiological nuances captured by each metric and underscoring the importance of selecting the most appropriate tool based on the clinical outcome of interest. This research fills a critical gap in the literature, which has historically relied on predominantly Caucasian cohorts and often focused on a limited number of indices, thereby providing evidence more directly applicable to the growing population of Chinese women with PCOS who exhibit distinct anthropometric and metabolic characteristics (8).

The identification of IR, a cornerstone defect in PCOS pathophysiology, remains a clinical priority. Our analysis revealed that LAP and CVAI were the most robust predictors, with near-identical AUCs of 0.815 and 0.813, respectively. The TyG index also demonstrated strong predictive capacity (AUC = 0.796). The outstanding performance of LAP can be attributed to its elegant simplicity, combining WC, a surrogate for central adiposity, with TG, a key marker of lipid dysregulation. This combination directly reflects the pathophysiological concept of “adipose tissue overflow,” where hypertrophied visceral adipocytes, unable to store more lipids, lead to increased lipolysis and elevated circulating free fatty acids (FFAs), which in turn promote ectopic fat deposition in liver and muscle and directly interfere with insulin signaling (25). Kahn initially proposed LAP as a marker of cardiovascular risk, but subsequent studies have validated its strong association with IR and hyperinsulinemia across diverse populations (20). A large cross-sectional study by Bozorgmanesh team confirmed that LAP was a stronger correlate of HOMA-IR than BMI or WC alone, aligning perfectly with our findings (26).

The CVAI’s performance is likely due to its more sophisticated, population-specific construction. Developed and validated for Chinese adults, CVAI incorporates age, BMI, WC, TG, and HDL-C, creating a composite score that captures not only the mass and distribution of adipose tissue but also its metabolic functionality—specifically, the dyslipidemic profile characterized by high TG and low HDL-C that is intimately linked to insulin resistance (19). The inclusion of age is particularly relevant, as insulin sensitivity naturally declines with age. A study by Xia demonstrated that CVAI was superior to VAI and other anthropometric indices in predicting IR in a general Chinese population, a conclusion our study now extends specifically to Chinese women with PCOS (27). The TyG index serves as a reliable and accessible surrogate for direct measures of insulin sensitivity. Its high performance underscores the close interplay between triglyceride metabolism and glucose homeostasis. Guerrero-Romero first established its strong correlation with the hyperinsulinemic-euglycemic clamp, the gold standard for measuring insulin sensitivity (21). The mechanism likely involves the sharing of common pathogenic pathways: insulin resistance impairs lipoprotein lipase activity, leading to elevated TG, while high TG and FFAs further exacerbate insulin resistance through the Randle cycle and pro-inflammatory pathways (28).

Traditional indices like WHR and WHtR remained significant but showed lower predictive performance for IR, with AUCs of 0.689 and 0.768, respectively. Notably, WHR and WHtR correlated most strongly with WC, which is mathematically inevitable because WC serves as the numerator in both formulas. This inherent correlation represents a limitation of the present study. Similarly, the associations of LAP with WC and TG may be influenced by its component variables. Nonetheless, these analyses were included to enable a comprehensive comparison of all indices and clarify their relationships with key metabolic parameters. Our findings reinforce that, although central adiposity is important, the metabolic profile reflected by lipid and glucose parameters confers stronger predictive power for IR in PCOS.

For the prediction of MetS, a cluster of cardiometabolic risk factors, the CMI, LAP, and VAI demonstrated exceptional diagnostic accuracy, with AUCs of 0.905, 0.898, and 0.893, respectively. The CMI, a relatively newer index, was the top performer. It is calculated as (WHtR) × (TG/HDL-C ratio), effectively integrating a measure of central adiposity relative to height with a powerful atherogenic lipid ratio. The TG/HDL-C ratio has been extensively validated as a strong marker of small, dense LDL particles, IR, and overall cardiovascular risk (29). Wakabayashi and Daimon, who proposed the CMI, found it to be a superior discriminator of diabetes and hypertension compared to other indices, a finding our study powerfully corroborates in the context of the full MetS spectrum in PCOS (22). The CMI’s strength lies in its simultaneous capture of anthropometric risk (via WHtR) and the resultant atherogenic dyslipidemia (via TG/HDL-C), which is a core component of MetS.

The equally impressive performance of LAP and VAI further solidifies the central role of lipid-overloaded visceral fat in driving MetS. VAI, similar to CMI, incorporates the TG/HDL-C ratio but uses WC and BMI in a gender-specific formula to estimate visceral adipose dysfunction. Amato et al. proposed VAI as a marker that reflects both the function and volume of visceral adipose tissue, which secretes a plethora of adipokines and cytokines that promote a pro-inflammatory, pro-atherogenic state (18). The convergence of high performance from CMI, LAP, and VAI sends a clear clinical message: the assessment of cardiometabolic risk in women with PCOS may be superior to the use of BMI, which has limitations in discriminating between fat and lean mass or accounting for fat distribution (30).

Beyond the core metabolic outcomes, our extensive correlation analyses reveal the profound systemic implications of adiposity in PCOS, linking these indices to reproductive, hepatic, renal, and even cardiac markers.

The strong and consistent negative correlations between all adiposity indices and SHBG, most strongly with CVAI (*r* = −0.542), are of paramount importance. Hepatic SHBG production is suppressed by hyperinsulinemia, which is a direct consequence of insulin resistance driven by visceral adiposity (31). Low SHBG increases the bioavailability of free testosterone, thereby amplifying clinical hyperandrogenism. This creates a vicious cycle where adiposity begets IR, which begets hyperandrogenism, which may further worsen metabolic parameters. Our data provide quantitative support for this pathophysiological model, directly linking adiposity indices to the core reproductive dysfunction in PCOS.

The positive correlations with liver enzymes ALT and AST, particularly with CVAI and LAP (*r* = 0.384 for both with ALT), reinforce the role of adiposity in driving subclinical hepatic injury, likely representing metabolic dysfunction-associated steatotic liver disease (MASLD—formerly non-alcoholic fatty liver disease, NAFLD). Furthermore, bilirubin fractions (TBIL, DBIL, and IBIL) were negatively correlated with all adiposity indices, suggesting a potential association between adiposity and systemic antioxidant capacity (32). However, the direct link between this correlation and metabolic abnormalities in PCOS, as well as its clinical significance, requires verification through further targeted studies.

Moderate weak correlations were observed between adiposity indices and partial renal indicators including B2-MG and CYSC, as well as myocardial enzymes LDH and CK-MB. In line with Table 2, B2-MG was significantly increased in the MetS group compared with the non-MetS group. These correlations may reflect the potential impact of adiposity on renal metabolism or myocardial function. However, since the primary outcomes of this study were IR and MetS, our study was not designed to assess organ dysfunction. Thus, we do not delve into the underlying mechanisms herein, and the relevant conclusions require further validation in subsequent studies.

This study’s strengths include a large, well-characterized cohort of Chinese women with PCOS from the PCOSAct trial and a comprehensive comparison of multiple adiposity indices for predicting insulin resistance and metabolic syndrome, supporting easy translation into clinical practice. Our study has several limitations that must be acknowledged. Firstly, this is a secondary analysis with a cross-sectional design, which precludes the establishment of causal relationships. Although we identified strong associations, we cannot determine whether elevated adiposity indices precede the development of IR and MetS. In addition, since the original PCOSAct trial was designed to evaluate the efficacy of acupuncture and clomiphene citrate for live birth in women with PCOS—not for validating adiposity indices—selective bias in variable collection may exist. Secondly, our study population exclusively included Chinese women with PCOS. While this strengthens internal validity for this specific demographic, it limits the generalizability of our findings to other ethnicities, PCOS phenotypes with milder metabolic disturbance, or male populations. Thirdly, several adiposity indices evaluated in this study share common components and are mathematically interrelated, which may lead to inherently strong correlations without independent biological implications; this statistical overlap should be considered when interpreting the correlational results. Fourthly, due to the lack of complete and standardized diagnostic data for MASLD, we were unable to evaluate the performance of adiposity indices in predicting MASLD in this cohort, which represents an important direction for future prospective studies with standardized liver assessments.

## 5 Conclusion

Our study provides robust, clinically relevant evidence that novel adiposity indices—especially CVAI, LAP, and CMI—are simple, non-invasive, cost-effective, and highly accurate tools for the early identification of major metabolic comorbidities in women with PCOS. These indices integrate routinely available clinical and biochemical parameters, going beyond the simplistic BMI paradigm to capture key dimensions of fat distribution and metabolic dysfunction. The study also proposes a potential clinical algorithm with CVAI or LAP preferred for screening IR and CMI or LAP optimal for assessing the risk of full-blown MetS. Their calculation can be easily integrated into electronic health records, providing clinicians with immediate, powerful risk scores to facilitate timely targeted interventions such as more aggressive lifestyle modifications, metformin therapy or earlier referral to hepatology or cardiology. Ultimately, this research aims to improve the long-term metabolic, hepatic, and cardiovascular health of women with PCOS, bridging the gap between metabolic risk identification and the implementation of effective preventive strategies in this high-risk population by translating complex pathophysiology into practical clinical tools.

## Data Availability

The raw data supporting the conclusions of this article will be made available by the authors, without undue reservation.
